# Supplementary material for: 2′FL and LNnT Exert Antipathogenic Effects against C. difficile ATCC 9689 In Vitro, Coinciding with Increased Levels of Bifidobacteriaceae and/or Secondary Bile Acids
Source: Pathogens. 2021 Jul 22;10(8):927. doi: 10.3390/pathogens10080927 (PMC8402123; doi:10.3390/pathogens10080927)
Supplement: Supplementary file 1 [file pathogens-10-00927-s001.zip › pathogens-1282460-supplementary.pdf]

pathogens

MDPI

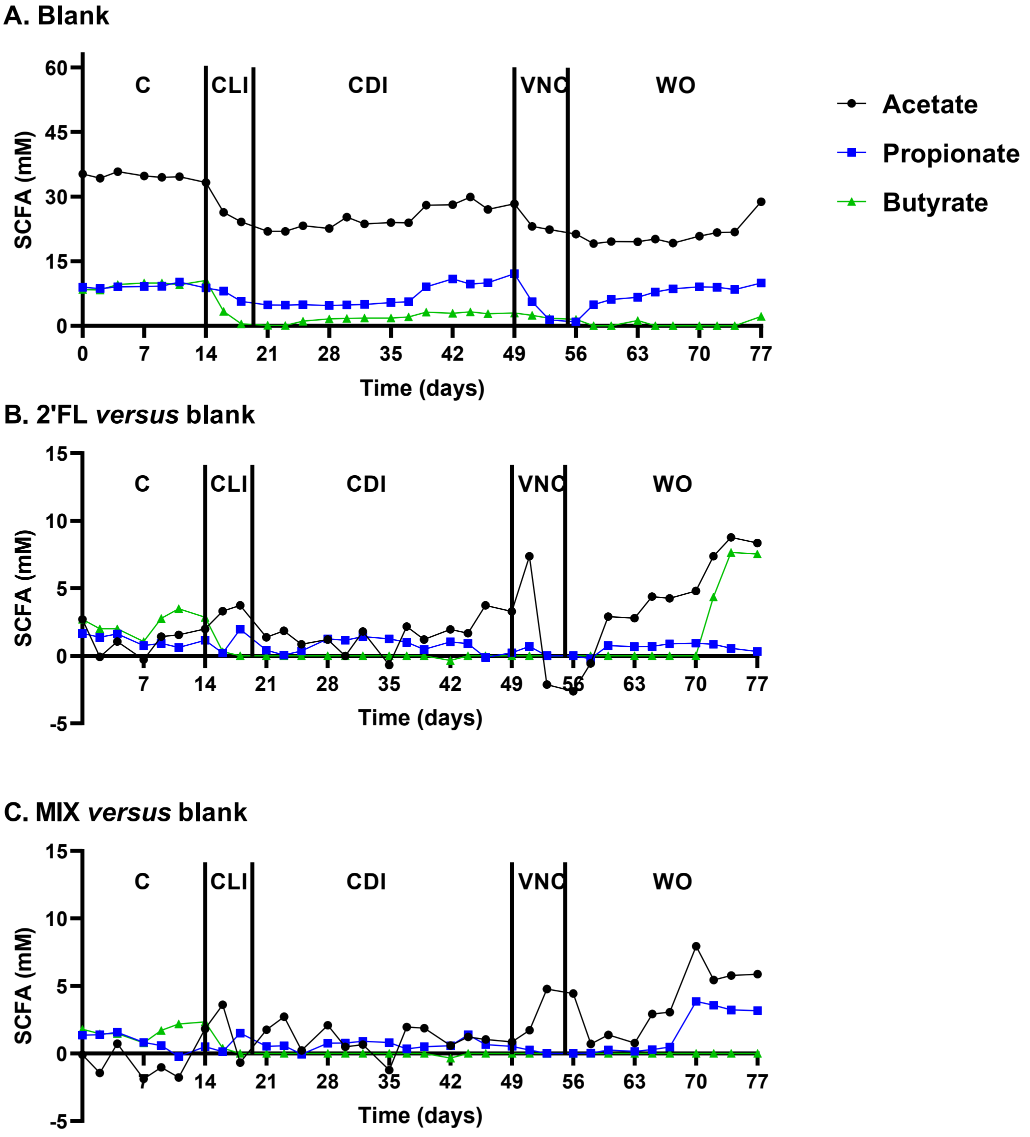

Figure S1. Acetate, propionate and butyrate levels (mM) in the proximal colon reactors during the long-term pathogut™ study (11 weeks) (test 2). Upon inoculation and stabilisation of the faecal inoculum of donor A during two weeks preceding the study (-14 – 0 days), a control period (0-14 days) was followed by a clindamycin treatment period (CLI: 14-21 days) which resulted in *C. difficile* infection (CDI: 21-49 days). From then on, while the blank was treated with vancomycin (VNC: 49-54 days), the other two arms additionally received 2'-FL and MIX, respectively. 2'FL and MIX were further administered during the washout period (WO: 54-77 days). While the absolute levels are presented for the blank reactor (**A**), the levels upon treatment with 2'FL (**B**) and MIX (**C**) are presented as the difference *versus* the blank reactor. 2'FL = 2'-O-fucosyllactose, LNnT = lacto-N-neotetraose, MIX = 4:1 mixture of 2'FL/LNnT.

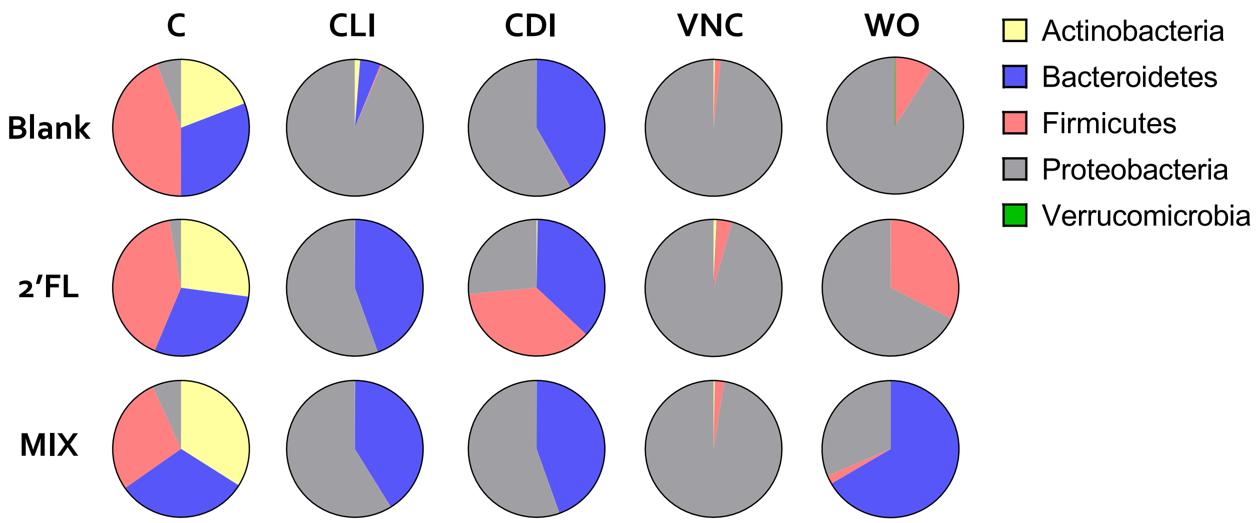

Figure S2. Microbial composition at phylum level in the distal colon reactors during the long-term pathogut™ study (11 weeks) as detected via 16S-targeted Illumina sequencing (test 2). Upon inoculation and stabilisation of the faecal inoculum of donor A during two weeks preceding the study (-14 – 0 days), a control period (0-14 days) was followed by a clindamycin treatment period (CLI: 14-21 days) which resulted in *C. difficile* infection (CDI: 21-49 days). From then on, while the blank was treated with vancomycin (VNC: 49-54 days), the other two arms additionally received 2′-FL and MIX, respectively. 2′FL and MIX were further administered during the washout period (WO: 54-77 days). 2′FL = 2′-O-fucosyllactose, LNnT = lacto-N-neotetraose, MIX = 4:1 mixture of 2′FL/LNnT.

pathogens MDPI

Table S1. Microbial composition at family level in the distal colon reactors during the long-term pathogut™ study (11 weeks) as detected via 16S-targeted Illumina sequencing (test 2). Upon inoculation and stabilisation of the faecal inoculum of donor A during two weeks preceding the study (-14 – 0 days), a control period (0-14 days) was followed by a clindamycin treatment period (CLI: 14-21 days) which resulted in *C. difficile* infection (CDI: 21-49 days). From then on, while the blank was treated with vancomycin (VNC: 49-54 days), the other two arms additionally received 2'-FL and MIX, respectively. 2'FL and MIX were further administered during the washout period (WO: 54-77 days). 2'FL = 2'-O-fucosyllactose, LNnT = lacto-N-neotetraose, MIX = 4:1 mixture of 2'FL/LNnT.

Phylum	Family	C			CLI			CDI			TR			WO		
		Blank	2'FL	MIX	Blank	2'FL	MIX	Blank	2'FL	MIX	Blank	2'FL	MIX	Blank	2'FL	MIX
Actinobacteria	Bifidobacteriaceae	12.12	25.27	21.06	0.02	0.15	0.35	0.00	0.10	0.00	0.00	0.02	0.01	0.00	0.00	0.00
	Coriobacteriaceae	0.36	0.27	0.29	0.00	0.01	0.06	0.04	0.03	0.02	0.00	0.00	0.00	0.00	0.00	0.00
	Micrococcaceae	0.00	0.00	0.00	0.00	0.00	0.00	0.01	0.00	0.00	0.13	0.07	0.14	0.01	0.01	0.00
Bacteroidetes	Bacteroidaceae	17.59	17.50	17.66	47.92	46.57	46.15	11.56	12.12	8.47	0.03	0.00	0.01	0.00	0.00	43.62
	Bacteroidales_S24-7_group	0.43	0.00	0.00	0.00	0.00	0.00	0.00	0.00	0.00	0.00	0.00	0.00	0.00	0.00	0.00
	Porphyromonadaceae	5.24	2.05	4.79	0.00	0.00	0.01	0.28	0.29	0.62	0.00	0.00	0.01	12.68	25.15	0.00
	Prevotellaceae	6.68	11.07	10.59	0.00	0.00	0.00	0.00	0.33	0.32	0.00	0.00	0.00	0.00	0.00	0.00
	Rikenellaceae	0.32	0.06	0.04	0.00	0.00	0.00	0.00	0.00	0.00	0.00	0.00	0.00	0.00	10.42	0.00
Firmicutes	Acidaminococcaceae	22.21	22.56	25.02	0.00	0.01	0.01	0.11	2.10	2.02	32.23	6.00	0.00	7.47	0.81	0.00
	Clostridiaceae_1	0.00	0.00	0.00	0.00	0.00	0.00	0.00	0.00	0.00	0.00	0.00	0.00	0.00	0.00	0.00
	Enterococcaceae	0.00	0.00	0.00	0.00	0.00	0.00	0.01	0.02	0.00	0.15	0.03	0.68	0.11	0.15	0.01
	Eubacteriaceae	0.19	0.11	0.04	0.00	0.00	0.00	0.03	0.03	0.00	0.00	0.00	0.00	0.00	1.33	0.00
	Lachnospiraceae	18.10	13.86	12.11	0.00	0.02	0.03	2.86	4.00	1.88	0.41	0.06	0.10	7.18	34.81	33.79
	Lactobacillaceae	0.00	0.00	0.00	0.00	0.00	0.00	0.00	7.67	0.00	0.00	0.03	5.16	0.00	0.67	0.03
	Paenibacillaceae	0.00	0.00	0.00	0.00	0.00	0.00	0.00	0.00	0.00	0.00	0.01	0.59	0.00	0.03	0.00
	Planococcaceae	0.00	0.00	0.00	0.00	0.00	0.00	0.00	0.00	0.00	0.00	0.00	0.02	0.00	0.00	0.00
	Ruminococcaceae	1.14	0.38	0.49	0.01	0.13	0.15	0.15	0.09	0.04	0.05	0.00	0.01	0.41	0.13	0.36
	Staphylococcaceae	0.00	0.00	0.00	0.00	0.00	0.00	0.00	0.00	0.00	0.07	0.00	0.00	0.00	0.00	0.00
	Veillonellaceae	3.59	1.05	1.27	0.00	0.00	0.01	0.00	0.00	0.00	0.00	0.00	0.00	0.00	0.00	0.00
Proteobacteria	Alcaligenaceae	1.27	0.42	0.72	0.00	0.01	0.08	0.17	0.07	0.02	1.22	9.76	2.32	0.35	0.11	0.01
	Brucellaceae	0.03	0.01	0.02	0.01	0.18	0.56	0.06	0.05	0.02	1.89	0.50	4.39	0.41	0.31	0.02
	Burkholderiaceae	0.00	0.00	0.00	0.00	0.01	0.01	0.00	0.00	0.00	0.01	0.00	0.00	0.00	0.00	0.00
	Desulfovibrionaceae	0.04	0.01	0.02	0.01	0.02	0.01	0.02	0.03	0.03	0.22	0.00	0.06	0.03	0.03	0.01
	Enterobacteriaceae	2.13	0.38	1.27	48.37	47.69	45.69	13.92	2.35	10.56	55.85	81.74	68.58	16.79	2.68	0.59
	Pseudomonadaceae	3.62	2.31	2.36	3.66	4.97	6.68	0.74	0.54	0.57	3.21	1.71	16.09	1.28	0.23	0.33
	Rhodospirillaceae	1.40	1.08	0.76	0.00	0.00	0.00	0.13	0.21	0.08	0.00	0.00	0.00	0.00	0.00	0.00
	Xanthomonadaceae	0.17	0.01	0.00	0.01	0.03	0.19	0.01	0.01	0.00	0.47	0.04	1.70	0.18	0.42	0.00
Verrucomicrobia	Verrucomicrobiaceae	2.32	1.57	1.48	0.00	0.00	0.01	69.32	69.95	75.34	4.02	0.03	0.11	53.06	22.72	21.22

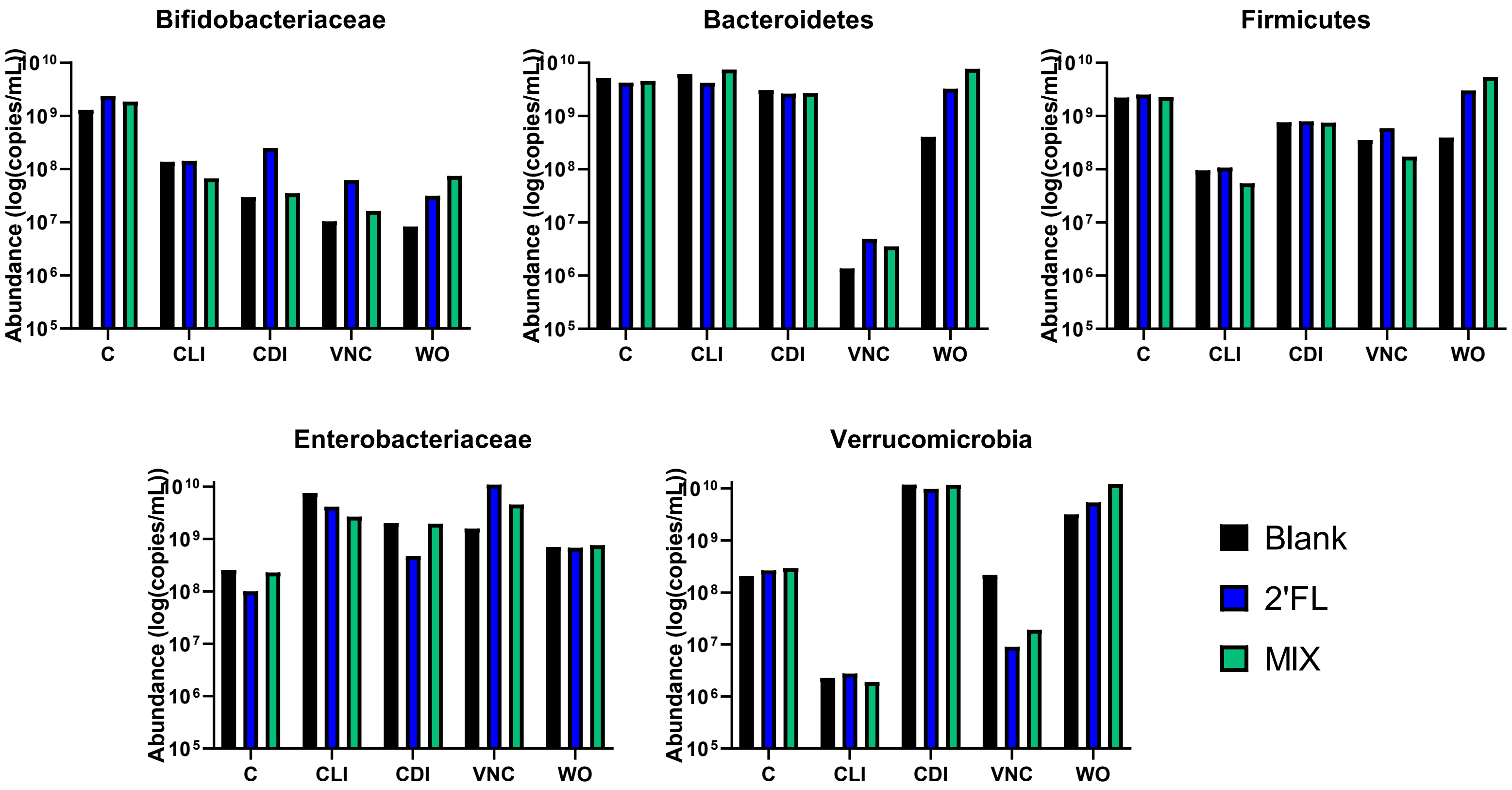

Figure S3. Microbial composition in the distal colon reactors during the long-term pathogut™ study (11 weeks) as detected via microbial group-specific qPCRs (test 2). Upon inoculation and stabilisation of the faecal inoculum of donor A during two weeks preceding the study (-14 – 0 days), a control period (0-14 days) was followed by a clindamycin treatment period (CLI: 14-21 days) which resulted in *C. difficile* infection (CDI: 21-49 days). From then on, while the blank was treated with vancomycin (VNC: 49-54 days), the other two arms additionally received 2′-FL and MIX, respectively. 2′FL and MIX were further administered during the washout period (WO: 54-77 days). 2′FL = 2′-O-fucosyllactose, LNnT = lacto-N-neotetraose, MIX = 4:1 mixture of 2′FL/LNnT.

pathogens

MDPI

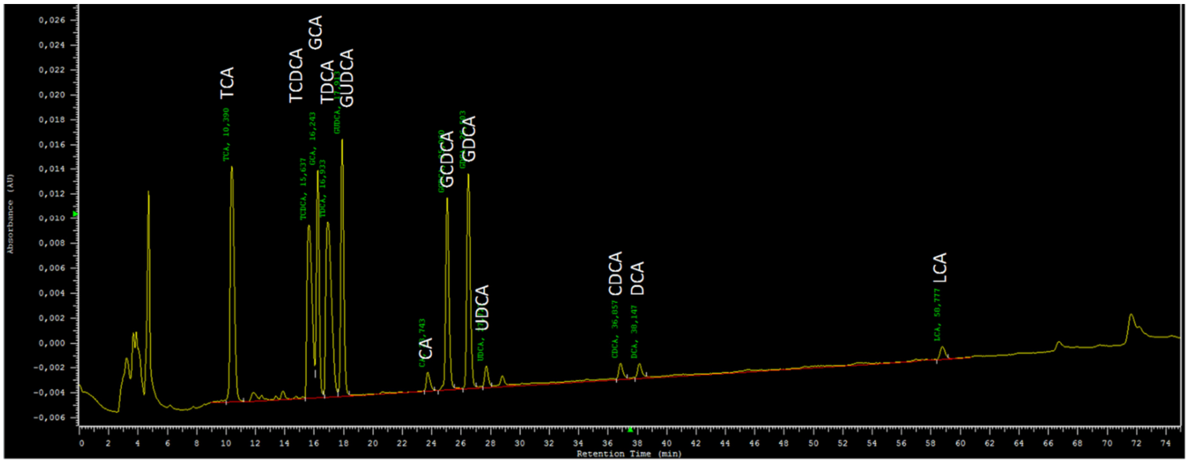

Figure S4. Chromatographic separation of twelve bile acids in one single run using reversed-phase HPLC-UV.
